# Anesthetic Management for Delivery in Parturients with Heart Disease: A Narrative Review

**DOI:** 10.3390/biomedicines13071736

**Published:** 2025-07-16

**Authors:** Shahab Ahmadzadeh, Drake P. Duplechin, Paris D. Bailey, Dillon T. Duplechan, Alexia J. Enache, Peyton Moore, Sahar Shekoohi

**Affiliations:** 1 Department of Anesthesiology, Louisiana State University Health Sciences Center at Shreveport, Shreveport, LA 71103, USA; 2 School of Medicine, Louisiana State University Health Sciences Center at Shreveport, Shreveport, LA 71103, USA; 3 School of Medicine, Louisiana State University Health Sciences Center at New Orleans, 433 Bolivar St, New Orleans, LA 70112, USA

**Keywords:** pregnancy, cardiac disease, obstetric anesthesia, cesarean section, hemodynamic changes, maternal outcomes, parturient

## Abstract

Cardiac disease remains a leading cause of maternal morbidity and mortality, particularly in developed countries where improved survival has increased the number of pregnant patients with congenital heart disease. The physiological changes of pregnancy, such as increased blood volume, cardiac output, and hypercoagulability, can exacerbate preexisting cardiac conditions, posing significant anesthetic challenges during cesarean delivery. This review outlines anesthetic strategies for parturients with structural or functional cardiac disease, emphasizing individualized, multidisciplinary care. We examine general and regional anesthesia approaches, intraoperative monitoring, and hemodynamic goals, including fluid balance, venous return optimization, and myocardial oxygen demand reduction. Preoperative risk stratification and coordination with cardiology and obstetric teams are essential. Future efforts should aim to standardize protocols and improve maternal–fetal outcomes through evidence-based anesthetic planning.

## 1. Introduction

The incidence of cardiovascular disease in pregnancy has grown steadily over the past several decades, influenced by increased maternal age, rising obesity rates, and improved survival of individuals with congenital heart disease into adulthood [1]. Cardiac disease has now become a leading cause of maternal mortality in high-resource countries, where advances in obstetric and perinatal care have minimized deaths from hemorrhage and infection [2]. For parturients with underlying heart disease, the physiologic demands of pregnancy and delivery can unmask latent dysfunction, precipitate decompensation, and significantly increase the risk of adverse maternal and fetal outcomes [3]. Pregnancy induces major cardiovascular adaptations that are necessary to meet the metabolic demands of the developing fetus and expanding uteroplacental circulation. These changes include a nearly 50 percent increase in circulating blood volume, an elevation in cardiac output, and a progressive decline in systemic vascular resistance [4]. Additionally, pregnancy is associated with a hypercoagulable state and structural changes in the heart, such as chamber dilation and left ventricular hypertrophy. While these changes are well tolerated in healthy women, they may destabilize individuals with valvular disease, pulmonary hypertension, cardiomyopathy, or ischemic heart disease [5]. In the peripartum period, abrupt hemodynamic shifts during labor, blood loss at delivery, and the sympathetic activation caused by pain and stress further increase cardiac workload, compounding the risk in vulnerable patients [6].

Anesthesiologists play a central role in managing the care of pregnant patients with heart disease, often beginning in the antepartum setting. Their responsibilities include participation in preoperative assessment, risk stratification, delivery planning, and intraoperative anesthetic management [7]. Selection of anesthetic technique, neuraxial or general, depends on the underlying cardiac lesion, anticipated hemodynamic response, and urgency of delivery [8]. Invasive monitoring, careful titration of medications, and multidisciplinary coordination are essential for optimizing maternal and fetal safety during delivery [9].

Despite the increasing prevalence of cardiac disease in pregnant patients, there remains no clear consensus on the optimal anesthetic technique for cesarean delivery in this high-risk population. Regional anesthesia is generally preferred due to its hemodynamic stability and avoidance of airway instrumentation, yet in patients with fixed cardiac output, severe valvular lesions, or reduced ejection fraction, the risk of hypotension and compromised perfusion raises significant concern. Conversely, general anesthesia may provide better control in hemodynamically unstable cases but introduces risks such as myocardial depression, intubation-associated stress, and delayed recovery. Both modalities, therefore, carry distinct risks depending on the underlying pathology, necessitating a highly individualized approach.

This lack of consensus is further complicated by a relative paucity of high-quality, stratified studies that compare outcomes across specific cardiac conditions. Most of the available literature is limited by small sample sizes, heterogeneous populations, and variable institutional practices, all of which hinder the development of standardized recommendations. Variation in provider expertise and anesthesiologist preference also contributes to inconsistency in clinical management. This review aims to synthesize existing evidence and provide practical guidance on anesthetic planning for cesarean delivery in patients with congenital or acquired heart disease, while identifying areas where further research is needed.

## 2. Pathophysiology of Cardiac Disease in Pregnancy

Pregnancy imposes complex cardiovascular adaptations that are essential for supporting fetal development, but they may prove deleterious for women with underlying heart disease [1]. One of the earliest and most dramatic changes is a near fifty percent increase in maternal blood volume, which begins in the first trimester and peaks in the third [4]. This volume expansion increases venous return and preload, leading to chamber dilation, particularly of the left atrium and left ventricle, which can overwhelm a compromised myocardium. In patients with restrictive cardiomyopathy or reduced ventricular compliance, this physiologic fluid load may result in pulmonary edema, exertional dyspnea, or frank heart failure [5]. Alongside preload augmentation, cardiac output increases by thirty to fifty percent, with stroke volume dominating early pregnancy and heart rate taking over in the later stages [3]. By term, heart rate may rise by ten to twenty beats per minute, a response that is typically benign but may unmask or worsen arrhythmias in susceptible women [2]. In cases of valvular stenosis, particularly mitral or aortic, increased cardiac output across a fixed orifice heightens transvalvular gradients, placing these patients at particular risk of pulmonary congestion and reduced perfusion [6]. Conversely, in valvular regurgitation, the fall in systemic vascular resistance that accompanies pregnancy may improve forward flow by decreasing afterload. SVR falls due to the vasodilatory effects of progesterone, nitric oxide, prostacyclin, and placental hormones. Although beneficial in maintaining uterine perfusion, this reduction in afterload may contribute to hypotension, especially in patients undergoing neuraxial anesthesia [5]. In hypertrophic obstructive cardiomyopathy, a sudden fall in SVR can worsen outflow tract obstruction and precipitate hemodynamic collapse [10]. After delivery, the rapid autotransfusion of blood from the contracting uterus and reversal of hormonal vasodilation causes abrupt increases in SVR, which may lead to acute decompensation if not anticipated [11].

Pregnancy is also characterized by a hypercoagulable state. Fibrinogen and clotting factors VII, VIII, IX, and X increase, while natural anticoagulants such as protein S decrease, and fibrinolytic activity is suppressed [8]. While these changes reduce hemorrhage risk at delivery, they significantly raise the incidence of venous thromboembolism and pose substantial danger to women with mechanical heart valves, who require careful anticoagulation management [9]. Balancing the bleeding risk of neuraxial techniques against the thrombotic risk of inadequate anticoagulation necessitates precise perioperative planning [10].

Respiratory changes in pregnancy include increased minute ventilation and oxygen consumption, accompanied by reduced functional residual capacity due to the upward displacement of the diaphragm [12]. These changes reduce maternal respiratory reserve and increase sensitivity to apnea and hypoxia during sedation or intubation, especially in women with coexisting pulmonary hypertension [13]. In pulmonary hypertension, the expected pregnancy-induced fall in pulmonary vascular resistance may not occur, and right ventricular failure becomes a leading cause of maternal death [14]. Monitoring oxygen saturation and avoiding excessive intravenous fluid administration are essential precautions in this group. Beyond the mechanical effects, the cardiovascular system in pregnancy is subject to hormonal and metabolic modulation that can unmask latent disease. For instance, increased estrogen may exacerbate some arrhythmogenic syndromes, while thyroid dysfunction, often seen in pregnancy, may provoke tachyarrhythmias or worsen heart failure in predisposed individuals [2]. Furthermore, anemia of pregnancy, if severe, can impose additional myocardial strain by reducing oxygen delivery, further elevating cardiac workload in women with reduced reserve [13]. Anesthetic implications of these pathophysiologic changes are profound. Careful preoperative assessment, including echocardiography, functional testing, and review of prior cardiac events, is needed to guide anesthetic technique [7]. General anesthesia may depress myocardial function or provoke hypertensive surges during laryngoscopy, while neuraxial anesthesia carries risks of abrupt preload reduction and hypotension. Preload optimization, vasopressor support, and invasive monitoring may all be necessary depending on the lesion and expected hemodynamic shifts. An individualized plan that aligns with the patient’s specific cardiac diagnosis, delivery mode, and anticipated physiologic challenges is essential [7].

Finally, the postpartum period demands continued vigilance. Many complications occur after delivery, particularly during fluid shifts and mobilization of peripheral edema [15]. Women with heart disease should be monitored closely for signs of decompensation and provided with anticipatory guidance regarding medications, activity, and follow-up care. Ultimately, success in managing these complex patients depends on multidisciplinary planning, detailed understanding of physiologic adaptations, and proactive, dynamic anesthetic care strategies [16].

## 3. Anesthetic Management Strategies

### 3.1. Preoperative Considerations

#### Risk Stratification and Pre-Delivery Planning

The modified World Health Organization (WHO) classification of maternal cardiovascular risk is a validated framework for assessing pregnancy-associated morbidity in women with underlying heart disease. This system stratifies patients into four classes based on the severity of their condition and the anticipated risk of pregnancy-related complications. Class I includes individuals with mild cardiac anomalies—such as mitral valve prolapse, isolated premature beats, or successfully repaired simple congenital defects—who are considered low risk and can be managed with routine obstetric care. Class II encompasses conditions like unrepaired atrial or ventricular septal defects, repaired Tetralogy of Fallot, or Turner syndrome without aortic dilation; these patients typically require periodic cardiology assessment throughout pregnancy. Class III signifies a substantially elevated risk and includes mechanical heart valves, systemic right ventricles, or moderately reduced ventricular function, necessitating multidisciplinary care in a tertiary center with expertise in high-risk pregnancies. Class IV represents conditions in which pregnancy is contraindicated due to an unacceptably high risk of maternal morbidity and mortality. This group includes severe pulmonary arterial hypertension, severely impaired systemic ventricular function (ejection fraction < 30%), and severe symptomatic valvular lesions such as critical mitral stenosis. Pregnancy is strongly discouraged in these cases. If pursued, intensive monitoring with monthly expert assessments is recommended throughout the pregnancy [14]. Given the high risk of complications associated with these conditions, preventative cardiovascular evaluations are essential. Cardiovascular disease is becoming increasingly common among pregnant women, partly due to the shift in childbearing age—from the early 20s to the 30s and 40s—and the rising use of in vitro fertilization [17]. Echocardiography is the most effective diagnostic tool for assessing cardiac disease in pregnant women due to its safety for both the mother and the baby. It provides detailed visualization of structural abnormalities within the heart and explains pathophysiological defects that could lead to serious complications. Echocardiography also aids in determining whether intervention is needed before or during pregnancy, as well as the severity of the cardiac disease. Given the physiological changes that occur during pregnancy, it is essential to distinguish between normal adaptations and potential signs of heart disease, which can be challenging to diagnose in this population [18]. Cases who are at higher risk for complications such as preeclampsia, hypertension, growth restriction, and other pregnancy-related outcomes should be assessed for cardiac function in early trimesters by echocardiography. This will help evaluate and determine maternal cardiac output and systemic cardiovascular resistance, consequentially being able to diagnose cardiac disease early. In the third trimester, because of the uterus compressing against the diaphragm, echocardiography makes it more difficult to evaluate cardiac functioning [19].

Pregnant patients with cardiovascular disease are managed by a multidisciplinary team known as the Pregnancy Heart Team. This team typically includes maternal–fetal medicine specialists, cardiologists, obstetric anesthesiologists, and neonatologists. These providers work collaboratively throughout the antepartum, intrapartum, and postpartum periods to develop and implement a comprehensive care plan tailored to the patient’s cardiovascular condition. Central to the success of this model is effective interprofessional communication and coordinated management, which are essential for optimizing both maternal and fetal outcomes [20].

### 3.2. Choice of Anesthesia: Regional vs. General

#### 3.2.1. Regional Anesthesia

There are three types of neuraxial anesthesia used for labor and delivery: epidural, spinal, and combined spinal–epidural anesthesia. Spinal anesthesia offers a rapid onset but may induce sudden hypotension, particularly in preload-dependent lesions. Epidural anesthesia allows for slower, titratable administration, providing hemodynamic stability but requiring additional time to reach surgical anesthesia. Combined spinal–epidural techniques may offer a compromise by balancing onset speed with control, but limited data exist on their comparative safety in cardiac parturients. Epidural anesthesia has the most gradual onset of medication effects, typically peaking 15 to 20 min after administration. It provides the greatest coverage in the sacral region compared to other types of neuraxial blockades used during labor [21]. A local anesthetic is injected near a group of nerves that transmit pain signals to provide pain relief during childbirth. Side effects may include hypotension, fever, urinary retention, and motor blockade. Studies have shown that epidural anesthesia does not increase the risk of cesarean section [22].

Spinal anesthesia is administered in the mid to lower lumbar region of the spine, where the anesthetic is injected into the subarachnoid space, which contains cerebrospinal fluid. It is typically indicated for relatively short procedures involving the abdomen, pelvis, or lower extremities. It has a relatively quick onset, low cost, and is achieved by a minimal volume of medication. Contraindications to spinal anesthesia include severe aortic or mitral stenosis and conditions causing left ventricular outflow tract obstruction, such as hypertrophic obstructive cardiomyopathy with associated pulmonary hypertension [23]. Careful anesthetic planning is essential in cardiac parturients to avoid decompensation during delivery, and guidance from the obstetric anesthesiology literature can support these decisions [24].

Combined spinal–epidural (CSE) anesthesia integrates both neuraxial techniques to optimize pain management during labor. It provides the rapid onset and potent analgesia of spinal anesthesia by delivering medication into the subarachnoid space, while also offering the flexibility and duration control of an epidural catheter. Doses can be titrated easily for individualized pain relief. Because epidural anesthesia typically requires a larger volume of anesthetic, combining it with spinal anesthesia may reduce the overall dosage needed and help minimize some of the side effects associated with epidural use alone [25].

#### 3.2.2. General Anesthesia

General anesthesia involves the administration of agents that induce unconsciousness in a patient, preventing the perception of pain and suppressing autonomic reflexes. There are five primary classes of anesthetic agents: intravenous (IV) sedatives, IV anesthetics, inhalational anesthetics, neuromuscular blocking agents, and opioids. Each plays a critical role in the induction and maintenance of anesthesia. Although neuraxial anesthesia is considered the gold standard and is relatively safe for most patients, general anesthesia may be necessary in certain situations. Absolute contraindications to neuraxial anesthesia include patient refusal, increased intracranial pressure, spinal cord injury, and local infection at the lumbar injection site. Relative contraindications include hemodynamic instability, thrombocytopenia, and obstructive cardiomyopathy [26]. Sympathetic blockade from neuraxial anesthesia reduces systemic vascular resistance and preload, necessitating close monitoring and fluid titration, particularly in those with fixed cardiac output lesions [27].

Intubation in pregnant patients is classified as a difficult airway due to anatomical and physiological changes associated with pregnancy. As such, the use of rapid sequence induction (RSI) agents is recommended, consistent with protocols used in non-pregnant patients. The incidence of failed intubation is notably higher in the obstetric population, which may be attributed to reduced exposure and training opportunities, given the declining use of general anesthesia in obstetric practice [28]. The selection of induction agents is guided by patient history, clinical status, and the nature of the surgical procedure. Commonly used agents include propofol, ketamine, and etomidate. Propofol is a lipophilic gamma-aminobutyric acid (GABA) agonist widely used for rapid sequence induction due to its potency, rapid onset, and short duration of action. It readily crosses the blood–brain barrier, achieving rapid central nervous system effects. The typical induction dose is approximately 1.5 mg/kg, adjusted according to patient weight. However, propofol may cause hypotension and bradycardia, particularly in cases with hypovolemia or reduced cardiac output [29].

Ketamine, also highly lipophilic, crosses the blood–brain barrier quickly and exerts its effects primarily through N-methyl-D-aspartate (NMDA) receptor antagonism. It provides both analgesia and amnesia and stimulates the sympathetic nervous system, leading to increased heart rate, blood pressure, and cardiac output. As a result, ketamine is often avoided in patients with severe heart failure, as it cannot compensate for the drug’s inherent negative inotropic effects. Etomidate is a sedative–hypnotic agent that enhances GABA receptor activity. It is valued for its rapid onset, brief duration of action, and minimal impact on cardiovascular stability, making it a suitable option in hemodynamically unstable patients [29].

When administering general anesthesia to obstetric patients, it is essential for anesthesiologists to understand the physiological changes associated with pregnancy. The upward displacement of the diaphragm by the enlarging uterus leads to a reduction in functional residual capacity, while minute ventilation increases by approximately 50%. During pregnancy, mucosal and laryngeal edema significantly contribute to the increased difficulty of tracheal intubation. These anatomic and respiratory alterations necessitate meticulous planning and preparation. A systematic, algorithmic approach to induction is critical for ensuring the safety and effectiveness of general anesthesia in this population [28]. Table 1 summarizes preferred anesthetic techniques for common cardiac conditions during delivery or cesarean section.

### 3.3. Intraoperative Monitoring and Hemodynamic Support

#### 3.3.1. Monitoring Strategies

Preoperative evaluation is essential for assessing surgical risk and formulating an individualized anesthetic plan prior to entering the operating room. Continuous monitoring of vital signs throughout the procedure is critical to allow prompt intervention by the anesthesia team in the event of physiological disturbances. Blood pressure serves as a key indicator of hemodynamic stability. In low-risk patients, intermittent monitoring using an automated oscillometric device is generally sufficient. However, in moderate- to high-risk patients or during complex surgical procedures, intra-arterial catheterization is often employed. Arterial line monitoring is considered the gold standard for real-time, accurate blood pressure measurement during surgery [32]. Central venous pressure (CVP) is another critical parameter for assessing hemodynamic stability, particularly in the context of fluid management. CVP reflects right atrial and right ventricular pressure, serving as an indirect measure of venous return, preload, and overall intravascular volume status. Measurement is achieved by the insertion of a central venous catheter, typically via the internal jugular or subclavian vein, with the catheter tip positioned in the superior vena cava or right atrium. Proper placement is confirmed radiographically, usually with a chest X-ray. CVP monitoring plays a vital role in perioperative assessment of tissue perfusion and guides volume resuscitation strategies [33,34].

In select high-risk surgical cases, transesophageal echocardiography (TEE) may be employed and interpreted by the anesthesiologist as an advanced intraoperative monitoring tool. TEE allows for detailed visualization of posterior cardiac structures that are often inadequately assessed by transthoracic echocardiography. Although not used for continuous monitoring, TEE provides an accurate real-time assessment of cardiac output, left and right ventricular function, hemodynamic instability, and shock. Furthermore, it is valuable for evaluating the efficacy of therapeutic interventions by comparing pre- and post-treatment cardiac performance [35]. TEE can also be used as a diagnostic tool intraoperatively. For example, in a patient undergoing surgery for severe mitral stenosis, preoperative TEE was performed to confirm the diagnosis. Given the elevated risk of thromboembolic events, intraoperative TEE was also conducted, which revealed the presence of a large left atrial thrombus. This highlights the diagnostic value of TEE in detecting critical cardiac pathology that may influence intraoperative management and surgical decision-making [36].

#### 3.3.2. Advanced Circulatory Support in High-Risk Cases

In patients requiring advanced cardiac hemodynamic support, the use of intra-aortic balloon pumps (IABPs) has demonstrated significant clinical benefits. IABPs function by inflating during diastole—synchronized with the second heart sound marking aortic valve closure—thereby reducing afterload and enhancing perfusion of both peripheral organs and coronary circulation. This mechanical support device improves diastolic pressure while simultaneously lowering systolic pressure, which reduces myocardial oxygen demand and ventricular wall stress. Clinical indications for IABP use include low cardiac output states and myocardial infarction complicated by left ventricular dysfunction. Evidence from recent studies suggests that both preoperative and perioperative implementation of IABPs is associated with reduced hospital length of stay, fewer ischemic events, and decreased incidence of renal failure [37]. Additionally, extracorporeal membrane oxygenation (ECMO) has emerged as a vital mechanical circulatory support strategy in critically ill patients. ECMO can provide systemic circulatory support when cardiac function is severely compromised or offer pulmonary support when respiratory failure is present. In peripartum cardiac, respiratory, or combined failure, venovenous or venoarterial ECMO is typically established using femoral vein and artery cannulation [38]. Although ECMO is associated with potential complications such as hemorrhage, thromboembolic events, and disseminated intravascular coagulation, its application during the peripartum and postpartum periods has been shown to yield favorable outcomes, with the benefits often outweighing the associated risks [39]. Finally, the left ventricular assist device (LVAD) represents a significant advancement in the management of patients with end-stage heart failure, particularly those awaiting heart transplantation. The device comprises several components, including an inflow cannula, a mechanical pump, an outflow cannula, a driveline, a controller, and an external battery pack. These elements work together to continuously circulate blood from the left ventricle directly into the aorta, thereby maintaining adequate systemic perfusion in patients with left ventricular dysfunction. In the obstetric population, the presence of an LVAD introduces substantial anesthetic challenges due to the physiological changes of pregnancy, including increased blood volume, decreased systemic vascular resistance, and altered cardiac output. As a result, meticulous preoperative planning is essential, often involving the use of transesophageal echocardiography (TEE) to evaluate cardiac function and optimize perioperative management. Intraoperative hemodynamic monitoring is critical, as anesthetic-induced reductions in preload may necessitate prompt intravascular volume resuscitation to maintain adequate device function and systemic perfusion. As the use of LVADs becomes more prevalent, their role in the multidisciplinary management of heart failure, including in pregnant patients, will continue to expand [40].

## 4. Clinical Findings: Case Analyses and Outcomes

### 4.1. Anesthetic Approach and Maternal Health

In a retrospective analysis of 3709 cesarean deliveries, 80% of patients received spinal anesthesia, while 20% underwent general anesthesia. The use of general anesthesia correlated with a higher rate of anesthesia-related complications and increased neonatal mortality [41]. A comprehensive study in Japan involving 89,225 women found that general anesthesia was linked to a 2.6-fold increase in severe maternal morbidity compared to neuraxial anesthesia, with higher odds of requiring red blood cell and plasma transfusions [42].

A meta-analysis encompassing 18 studies with 1109 patients suffering from pulmonary arterial hypertension indicated that regional anesthesia significantly reduced maternal morbidity, leading to lower postoperative blood pressures, shorter ICU stays, and decreased maternal mortality [43]. Similarly, research involving 220 patients with valvular disease concluded that neuraxial anesthesia was the preferred and safer technique, with no cases where the severity of cardiac disease precluded the use of regional anesthesia [44].

Despite these findings, maternal cardiac complications can still occur. In a study of 83 parturients with valvular heart disease, 9.64% experienced maternal cardiac events, including one death, even though regional anesthesia was used in 62.1% of cesarean sections. These outcomes underscore the necessity for thorough risk assessment and individualized care planning [45].

Developing individualized anesthetic plans for parturients with heart disease is necessary to optimize maternal and neonatal outcomes. As shown in Table 2, anesthetic plans should be developed on an individualized basis. Numerous factors should play into the development of an anesthetic plan. For example, mothers who are medicinally anticoagulated are unable to receive spinal anesthesia and should receive general anesthesia for cesarean sections [46]. For high-risk cardiac patients, the anesthetic goal should be centered around the patients’ cardiac conditions to minimize risks. For example, in patients with left-to-right cardiac shunts, the goal should be to minimize unnecessary increases in vascular resistance to minimize the strain on the right ventricle. Another example is with mitral stenosis, in which the goal should be to provide adequate analgesia to reduce any tachycardic response to pain [46].

### 4.2. Case Analyses: Customized Anesthetic Strategies

Individual case reports illustrate the importance of tailoring anesthetic approaches to specific cardiac pathologies. One case involved a 30-year-old woman with severe rheumatic heart disease, including significant mitral regurgitation and moderate pulmonary hypertension. She underwent an elective cesarean section using spinal anesthesia and bilateral transversus abdominis plane (TAP) blocks. Although initial hypotension occurred intraoperatively, it was managed effectively, resulting in favorable maternal and neonatal outcomes. The mother remained stable throughout the postoperative period and was discharged after 4 days [47].

Another report described two high-risk cases involving thoracic aortic aneurysms. One case presents a 33-year-old primigravida twin pregnancy with thoracic aortic aneurysm who underwent a cesarean section. The other case presents a 37-year-old with a heterozygous pathogenic variant of FLNA (filamin A), diagnosed with mild mitral and aortic regurgitations and a mild ascending aortic aneurysm, who also underwent a cesarean section. General anesthesia was judiciously chosen for both cases to control the sympathetic response to intubation and maintain hemodynamic stability, critical to preventing aortic dissection. Both cases resulted in stable intraoperative courses, smooth recovery in the cardiothoracic ICU, and hospital discharge by postoperative day five [48].

A further case describes a 24-year-old primigravida with twin gestation who was diagnosed with peripartum cardiomyopathy complicated with preeclampsia. Preanesthetic examination showed dilated cardiac chambers and global hypokinesia with a left ventricular ejection fraction of 25%. Due to the patient’s condition, anesthesia was given via a combined spinal–epidural technique. The patient underwent a cesarean section, in which a dead fetus and a living neonate were delivered uneventfully. Postoperatively, the mother was transferred to the ICU for further monitoring. On postoperative day 6, the mother was transferred to the cardiac center to undergo temporary placement of a pacemaker to treat resistant bradyarrhythmia. The report does not mention any immediate postpartum complications to the living baby, but it does state that continuous follow-up with the cardiac center by the researchers found that the baby continued to do well. The mother continued to have features of moderate ventricular dysfunction and was advised against any future pregnancies [49].

### 4.3. Neonatal Health Outcomes

Neonatal outcomes are also influenced by the choice of anesthetic technique. A comparative study of 287 cesarean deliveries found a significantly higher incidence of 5 min Apgar scores < 7 in neonates born to mothers who received general anesthesia compared to spinal anesthesia [50]. A 5 min Apgar score < 7 with the use of general anesthesia shows that the deleterious effects of the anesthesia may last longer than expected [49]. Researchers concluded that spinal anesthesia is recommended for elective cesarean sections due to decreased risk of neonatal depression [49,51].

In a cohort of 119 women with various cardiac diseases, 24% of neonates experienced adverse outcomes, primarily due to low birth weight, preterm birth, and fetal demise. In the study, 16% of deliveries were preterm, with 47% of those being due to maternal cardiac complications [52]. Another study found that mild congenital heart disease increased the risk of preterm delivery by 1.3-fold, and moderate to severe CHD increased this risk 3-fold, with correspondingly lower birth weights [53].

Neonatal intensive care unit (NICU) admission is a critical factor when planning anesthesia for pregnant women with heart disease, especially in preterm deliveries. One study reported that 19.4% of neonates born to mothers with heart disease required NICU care, with a significant proportion being preterm [54]. As depicted in Table 2, the anesthesia plan should be made on a case-by-case basis to maximize both maternal and neonatal outcomes. Therefore, anesthesia planning should examine all aspects of the mother’s well-being, gestational age, and the well-being of the fetus.

### 4.4. Key Takeaways

Collectively, these findings suggest that regional anesthesia is generally preferred for cesarean delivery in patients with heart disease due to lower maternal morbidity and improved neonatal outcomes. While general anesthesia may be necessary in certain situations—such as anticoagulation, connective tissue disorders, or aortic pathology—it should be employed with careful planning and intensive monitoring. Table 2 indicates the importance of individualized anesthesia plans based on the mother’s predisposing conditions to improve both maternal and neonatal outcomes. The risk of maternal cardiac decompensation and adverse neonatal outcomes increases in cases of prolonged labor, preterm delivery, and severe cardiac pathology, emphasizing the need for individualized care plans and multidisciplinary management.

## 5. Conclusions

The anesthetic management of pregnant patients with cardiac disease is a critical area that requires attention due to the complexities involved. As the incidence of cardiac conditions in pregnant women rises, understanding the nuanced anesthetic considerations becomes increasingly important for both maternal and fetal safety. Key findings from this discussion emphasize the necessity of individualized anesthetic planning, highlighting that regional anesthesia is typically favored but should always be adapted according to the patient’s specific cardiac pathology. Additionally, advanced monitoring techniques and circulatory support may be essential in managing severe cases, ensuring that the unique physiological changes during pregnancy are adequately addressed. The significance of these findings lies in their potential to enhance outcomes for high-risk parturients, ultimately leading to safer deliveries and improved maternal health. As we move forward, there is a clear need for prospective trials to evaluate anesthetic techniques in these complex cases, as well as exploration of emerging cardiac interventions that could further optimize maternal outcomes. In conclusion, the anesthetic care plan of pregnant patients with cardiac disease should be constructed to address the needs and minimize the risks of each individual patient. Additionally, it is generally accepted that regional anesthesia is preferred over general anesthesia, but general anesthesia may be required based on the needs and comorbidities of the patient.

## Figures and Tables

**Table 1:** Summary of cardiac condition.

Cardiac Condition	Preferred Anesthetic Technique	Technique to Avoid	Comparison/Clinical Rationale
Mitral Stenosis [21]	Epidural anesthesia Maternal and neonatal outcomes were uneventful	General anesthesia is typically used when neuraxial is contraindicated; ephedrine should be avoided	Low fixed cardiac output and fluid control; avoids increase in heart rate
Aortic Stenosis [21]	Slow titration of epidural anesthesia Patient recovered without complication following neuraxial anesthetic	General anesthesia is typically used when neuraxial is contraindicated; volatile anesthetics are CI (isoflurane, sevoflurane)	Low fixed cardiac output and fluid control; avoids vasodilation and tachycardia
Dilated Cardiomyopathy (DCM) [30]	Epidural anesthesia Maternal and neonatal outcomes were uneventful	Volatile anesthetics	Avoid increases in afterload and decreases in myocardial depression
Hypertrophic Cardiomyopathy (HCM) [31]	Epidural anesthesia w/precise drug titration Hemodynamic stability was maintained throughout the procedure	Spinal anesthesia, ephedrine, dobutamine, and dopamine	Decrease in preload and increase in contractility worsen obstruction; avoids hypotension and tachycardia
Pulmonary Hypertension (PH) [21]	General anesthesia Despite transient hypotension, overall outcomes remained stable	Neuraxial blockade and nitrous oxide	Avoids agents that increase pulmonary vascular resistance

**Table 2:** Comparative studies.

Cardiovascular Disease	Anesthetic Approach	Maternal Outcome	Fetal Outcome
Severe rheumatic disease with mitral valve regurgitation and moderate pulmonary hypertension [46]	Spinal anesthesia with bilateral transabdominal plane blocks Hemodynamic stability was maintained throughout the procedure	No complications; discharged 4 days post-op	No complications
Ascending aortic aneurysm [47]	General anesthesia Patient recovered without complications following neuraxial anesthetic	No complications; discharged 5 days post-op	No complications
Mild mitral and atrial valve regurgitation with mild ascending aneurysm [47]	General anesthesia Despite transient hypotension, overall outcomes remained stable	No complications; discharged 5 days post-op	No complications
Severe aortic stenosis [48]	Combined spinal–epidural anesthesia Maternal and neonatal outcomes were uneventful	No intraoperative complications; postoperative left lower extremity muscle weakness due to epidural catheter dislodgement; loss of epidural catheter placement on day 2 post-op; no other complications; discharged 7 days post-op	No complications
